# MXene/PPy@PDMS sponge-based flexible pressure sensor for human posture recognition with the assistance of a convolutional neural network in deep learning

**DOI:** 10.1038/s41378-023-00605-0

**Published:** 2023-12-19

**Authors:** Hui Xia, Lin Wang, Hao Zhang, Zihu Wang, Liang Zhu, Haolin Cai, Yanhua Ma, Zhe Yang, Dongzhi Zhang

**Affiliations:** 1 College of Control Science and Engineering, China University of Petroleum (East China), Qingdao, 266580 China; 2 State Key Laboratory of Chemical Safety, SINOPEC Research Institute of Safety Engineering Co., Ltd, Qingdao, 266071 China

**Keywords:** Nanosensors, Sensors

## Abstract

The combination of flexible sensors and deep learning has attracted much attention as an efficient method for the recognition of human postures. In this paper, an in situ polymerized MXene/polypyrrole (PPy) composite is dip-coated on a polydimethylsiloxane (PDMS) sponge to fabricate an MXene/PPy@PDMS (MPP) piezoresistive sensor. The sponge sensor achieves ultrahigh sensitivity (6.8925 kPa^−1^) at 0–15 kPa, a short response/recovery time (100/110 ms), excellent stability (5000 cycles) and wash resistance. The synergistic effect of PPy and MXene improves the performance of the composite materials and facilitates the transfer of electrons, making the MPP sponge at least five times more sensitive than sponges based on each of the individual single materials. The large-area conductive network allows the MPP sensor to maintain excellent electrical performance over a large-scale pressure range. The MPP sensor can detect a variety of human body activity signals, such as radial artery pulse and different joint movements. The detection and analysis of human motion data, which is assisted by convolutional neural network (CNN) deep learning algorithms, enable the recognition and judgment of 16 types of human postures. The MXene/PPy flexible pressure sensor based on a PDMS sponge has broad application prospects in human motion detection, intelligent sensing and wearable devices.

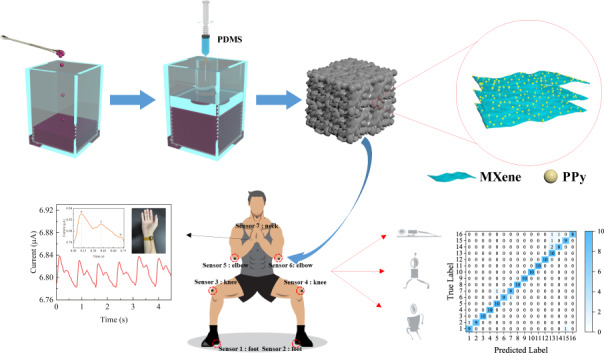

## Introduction

Flexible pressure sensors have attracted great attention because of their detection capabilities and wide application prospects^1–3^. Moreover, such devices can be attached to various key parts of the body to monitor various activities. To date, various categories of pressure sensors, such as capacitive^4–6^, piezoresistive^7–9^, piezoelectric^10–12^, and triboelectric^13–15^ sensors, are beginning to be used in the next generation of wearable electronic devices. Among these categories, pressure sensors based on the piezoresistive effect have the advantage of easy signal processing and dynamic sensing^16^. Ding et al. constructed flexible piezoresistive sensors with higher sensitivity and better stability by modulating sensitive materials and designing microstructures^17^. However, low sensitivity, poor flexibility, slow response/recovery and narrow measurement range still limit the practical application of flexible pressure sensors. Therefore, establishing more sensitive materials and advanced technologies for the fabrication of high-performance flexible pressure sensors remains a challenge.

Currently, typical substrate materials for piezoresistive sensors include hydrogel^18–21^, polydimethylsiloxane (PDMS)^22–24^, and sponge^25–28^. Yang et al. used aerogels to prepare flexible sensors, but these materials are especially difficult to apply because of their high production cost and irreversible deformation^29^. Contrary to the rigid characteristics of traditional pressure sensing materials, sponges have gained increasing attention due to their good flexibility and high porosity^30^. PDMS is often further broadened by structural design due to its flexibility and ease of preparation^31^. In this work, the use of a PDMS sponge as the substrate of the sensor provides the possibility for its large-scale preparation.

MXene is currently one of the most promising materials in the sensor field due to its excellent electromechanical properties, abundant active sites, and good hydrophilicity^32–34^. The 2D transition metal carbide and nitride family (MXene, denoted as M_n+1_X_n_T_x_, where M denotes transition metal, *n* = 1, 2 or 3, X is C and N or N, while T denotes surface functional group) exhibit fine mechanical strength and superior metallic conductivity. However, in air, oxygen attacks the Ti atoms in MXene to oxidize it, which affects the electrical conductivity. Therefore, the key to improving the stability of MXenes depends on blocking the contact between oxygen and Ti atoms. Conductive polymers have become exciting research materials in sensing, energy storage, electromagnetic shielding, and metal corrosion because of their outstanding properties^35,36^. Common conductive polymers include polypyrrole (PPy)^37^, polythiophene (PTH)^38^, and polyaniline (PANi)^39^. Among them, PPy has remarkably good conducting properties. However, it presents difficulty when dissolving in organic solvents, which causes difficulties in the preparation of subsequent sensors. By an in situ polymerization method, PPy can form a continuous coating or film on many types of materials, thereby solving the problem of PPy agglomerating in solution and its difficulty in adhering to sensors. The in situ polymerization and uniform deposition of pyrrole nanoparticles in the MXene solution resulted in a protective Ti atomic coating of pyrrole. Meanwhile, the binary self-assembly of MXene nanosheets and PPy nanoparticles increases the conductive contact sites within the sensing layer, thus improving the conductivity of the sensor.

Here, we prepared MXene/PPy@PDMS (MPP) sponges by dipping-coating an MXene/PPy composite solution with a PDMS sponge to construct flexible pressure sensors. The PDMS sponge was prepared by the sacrificial sugar template method, and a composite MXene/PPy solution was prepared by in situ polymerization of PPy adsorbed on an MXene solution. This flexible pressure sensor achieves short response and recovery times and of 100 ms and 110 ms, respectively. It also exhibits a good piezoresistive response with a sensitivity of 6.8925 kPa^−1^ in 0–15 kPa and a maximum measuring range of 0.43 Pa–275 kPa. In addition, the effectiveness of the MPP sensor in detecting the movement of various joint parts of the body was investigated. A convolution neural network (CNN) deep learning algorithm was used to analyze the movement data and further recognize different kinds of human postures. Accordingly, the capabilities of MPP sensors in the fields of health monitoring and artificial intelligence were demonstrated.

## Experimental

### Material

PDMS and curing agent were obtained from Dow Corning, USA. Pyrrole (C_4_H_5_N), ferric chloride hexahydrate (FeCl_3_-6H_2_O), hydrochloric acid (HCl, analytical purity), and lithium fluoride (LiF, ≥99%) were provided by Sinopharm Chemical Reagent Co. Titanium aluminum carbide powder (Ti_3_AlC_2_, 98%, 200 mesh) and dodecylbenzene sulfonic acid (DBSA) were obtained from Shanghai Maclean Biochemical Technology Co. All chemicals were available without further purification. Deionized water was applied throughout the experiment.

### Preparation of the MXene/PPy composite

Preparation of the MXene/PPy composite solution mainly includes two stages. The first stage is the preparation of the MXene nanosheet solution. The preparation approach is based on the etching method we employed before^40^. The specific procedure is as follows. Two grams of LiF was added to 20 ml of hydrochloric acid (9 M) and stirred for 5 min to dissolve it. Two grams of Ti_3_AlC_2_ was slowly added and stirred thoroughly at 35 °C for 24 h to ensure that the force between the Al layer and Ti_3_C_2_ was weakened so that the Al layer could be peeled off more easily. The etching reaction products were centrifuged at 3500 rpm for 5 min, and the supernatant was poured out. This process was repeated several times until the pH of the supernatant was >6. The purpose is to ensure that the byproducts of the etching reaction are completely washed away to obtain MXene nanosheets.

The second stage is the adsorption of PPy nanoparticles on MXene nanosheets. DBSA and pyrrole monomer were stirred for 1 h in an ice water bath. Then, 40 ml of 10 mg/ml MXene solution was added. The addition of DBSA served to increase the electrical conductivity of PPy, enabling it to be deposited more adequately on MXene nanosheets by electrostatic adsorption. After 1 h, FeCl_3_-6H_2_O was added and stirred in an ice water bath for 6 h. During that period, the pyrrole monomer commenced to self-polymerize on the surface of the MXene nanosheets and form a continuous conductive layer. Finally, the reaction product was centrifuged several times to remove DBSA and FeCl_3_ to obtain the MXene/PPy composite solution. In addition, the ratio of MXene to PPy in the composite solution was controlled by adding different volumes (0.2, 0.3, 0.4, 0.5, and 0.6 mL) of pyrrole monomer.

### Preparation of the MPP sensor

**Fig. 1:**
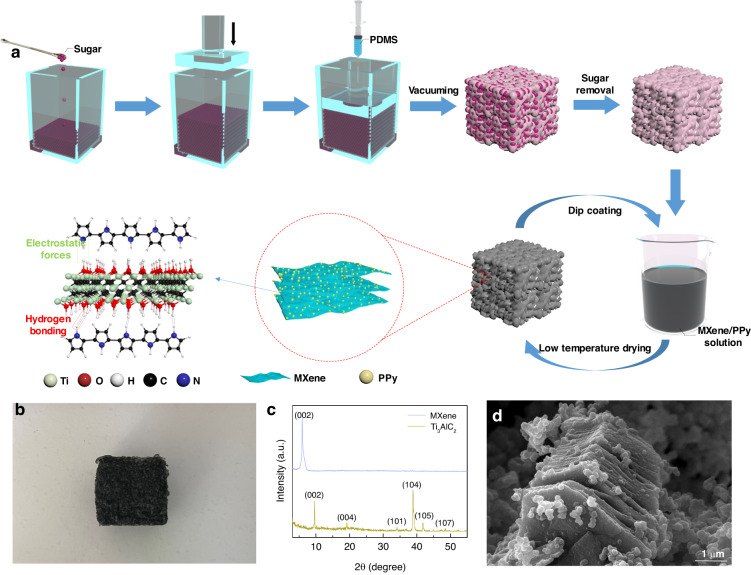
The preparation process and characterization of the MPP sensor. **a** Preparation process of MPP sponge as well as microscopic and molecular morphology schematic of MXene/PPy. **b** The MPP sponge digital image graph. **c** XRD patterns of MXene and Ti_3_AlC_2_. **d** SEM image of the MXene/PPy composite materials

PDMS sponges were prepared by the sacrificial sugar template method. As shown in Fig. 1a, white granulated sugar was placed in a hexahedral container with an open top of 2 cm in length and width. These sugar granules were compressed into regularly shaped cuboids (2 cm × 2 cm × 1 cm), and then the PDMS and curing agent mixture was injected into the container at a ratio of 10:1. After 2 h of vacuum drying, the pores inside the sugar cube were filled with the PDMS mixture. When the PDMS was cured, porous PDMS sponges of the same size as the original sugar cube were obtained by immersing the sugar/PDMS cuboid in water to remove the white granulated sugar. MPP sponges were prepared by coating MXene/PPy composites on a PDMS sponge skeleton by solution impregnation and low-temperature drying. The hydrogen bonds formed by the hydrogen atoms on PPy and the hydrogen atoms on MXene and the electrostatic interaction between PPy and MXene together led to a large intermolecular force between the MXene/PPy composites for stable adsorption (bottom left of Fig. 1a). An image of the MPP sponge is shown in Fig. 1b. For comparison, MPP sponges with different material ratios (as the concentration of PPy increased, the sponge was defined MPP-1–MPP-5), MPP sponges with different porosities, MXene@PDMS (MP) sponges and PPy@PDMS (PP) sponges were additionally fabricated by the same preparation process.

## Results and discussion

### Characterization of the MPP sensor

The phase analysis of the as-prepared materials was performed by X-ray diffraction (XRD, Rigaku D/Max 2500PC). As shown in Fig. 1c, after etching by HCl/LiF, the peaks at 34.0° (101), 38.8° (104), 41.7° (105), and 48.4° (107) distributed on Ti_3_AlC_2_ disappear in the MXene mode. Moreover, peak broadening and a negative shift are observed on the (002) crystal plane, indicating that Ti_3_AlC_2_ was etched out of the Al layer and successfully converted to MXene. The morphology of the samples was characterized by scanning electron microscopy (SEM, Hitachi S-4800, Japan), which was performed at 10 kV and 15,000x. As shown in Fig. 1d, the MXene presents an accordion-like multilayer structure with PPy nanoparticles (particle size <1 μm) attached to its surface. The SEM images of MPP sponges with different porosities prepared by different particle sizes (344, 544, 910 µm) of sugar are shown in Fig. S1.

### Mechanical properties of the MPP sensor

**Fig. 2:**
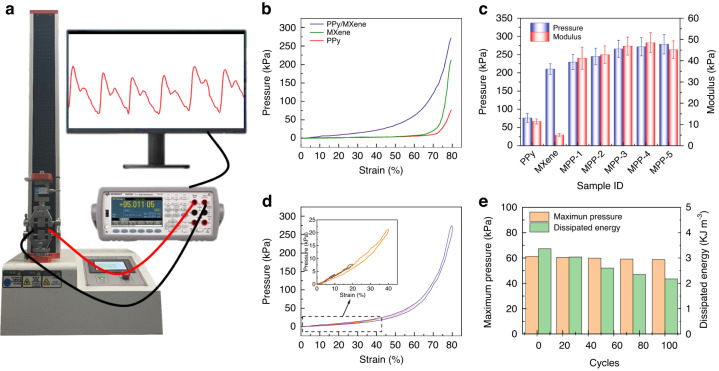
Mechanical testing of MPP sensors. **a** Schematic diagram of the piezoresistive response sensing platform. **b** Compressive stress‒strain curves of MPP, MP and PP sponges. **c** The pressure and modulus of PPy, MXene and MPP sponges at 80% compression strain. **d** Cyclic stress‒strain curves of the MPP sponge. **e** Maximum stress and dissipated energy of the MPP sensor at 1–100 repetitive stress strains

Evaluation of the mechanical properties of flexible pressure sensors is essential because it is necessary to maintain good flexibility along with superior mechanical properties. Figure 2 shows the mechanical properties test of the sponge sensors. The comprehensive performance for stress‒strain tests of the devices is performed by a ZQ-900A tensile tester, Agilent data acquisition instrument (B2902A) and computer (Fig. 2a). Figure 2b shows the compressive stress‒strain curves of the MPP sponge, MP sponge and PP sponge. The MP and PP sponges exhibited compressive pressures of 20.7 and 10.9 kPa under 70% compression strain. MPP sponges with MXene and PPy composites exhibit a compressive stress enhancement of 121.74 kPa, and the combination of MXene nanosheets and PPy nanoparticles promotes effective energy dissipation in the device, thereby improving the mechanical strength and toughness of the sponge. Figure 2c shows the compression performance of MP, PP sponges and MPP sponges with different material ratios at 80% strain. The data clearly show that the compressive strength and modulus of the composite sponge sensors are superior to those of the single material sponges, and the higher the concentration of PPy is, the higher the compressive strength of the composite sponges. This occurs mainly due to the layer-sphere support architecture between MXene and PPy, which allows for effective load transfer upon compression. However, an excessively high PPy concentration in turn decreases the modulus of the composite sponge due to the excessive accumulation and shedding of PPy nanoparticles. Figure 2d shows the cyclic compression stress‒strain curves of the MPP sponge at 10, 20, 40, 60, and 80% strain. The curves can generally be divided into two phases. The first stage is 0–40% strain (inset of Fig. 2d), when the stress and strain are almost linear. This occurs due to the large number of pores inside the sponge at this stage. The stress mainly depends on the supporting force of the sponge skeleton. The second stage is 40–80% strain, where the stress and strain are nonlinear. The reason is that most of the pores inside the sponge disappear at this point, and the sponge skeleton begins to contact each other. In addition, the stress‒strain curves during compression and release basically coincide, and the maximum hysteresis of the MPP sponge is only 10% (Fig. S2).

Figure S3 shows the stress‒strain curves of 100 consecutive compressions at 60% strain. The pressure of the MPP sensor only decreases slightly with an increasing number of cycles, and the curves corresponding to different cycles almost overlap. The above results prove that the porous PDMS sponge prepared by the sacrificial sugar template method has satisfactory stability. The MPP sensor with 100 compression stress‒strain is further analyzed (Fig. 2e). The maximum pressure at which the sensor attains 60% strain (61.23 kPa at the first cycle and 58.74 kPa at the hundredth cycle) does not change significantly with the number of cycles. The decrease in stress is attributed to the stress relaxation in the sponge skeleton. In addition, the MPP sensor still exhibits a high dissipation energy of 0.02174 kJ m^−3^ after 100 compressive stress‒strain cycles, which further demonstrates the excellent anti-fatigue and repetitive stability of the structure.

Six identical MPP sponges were fabricated by the same preparation method, and stress‒strain tests were carried out on the six sponges under equivalent conditions. All six sensors exhibit a nearly homogeneous stress response (the maximum difference between devices is only 4%) at large strains (70%) and small strains (5%) (Figs. S4 and S5), indicating the potential for large-scale preparation and standardized production of MPP sensors.

### Electrical properties of the MPP sensor

**Fig. 3:**
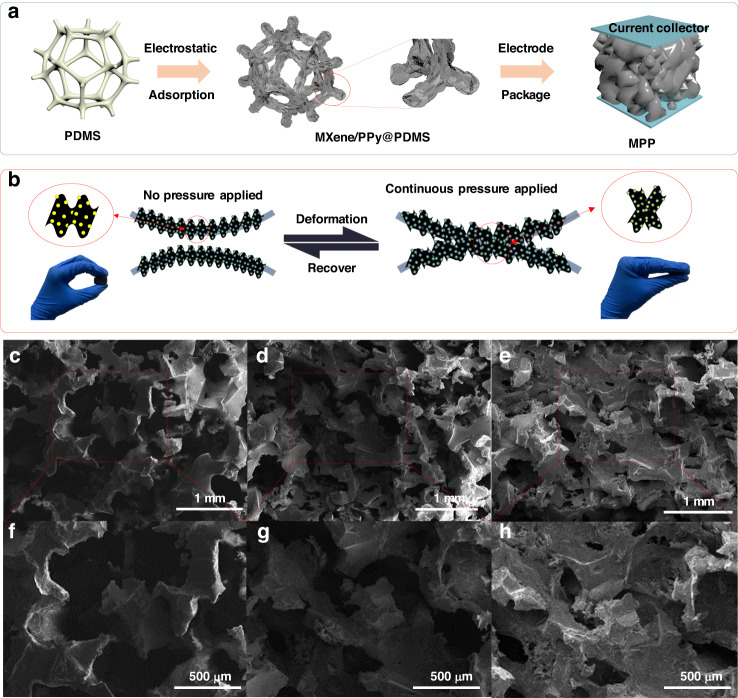
Mechanism of preparation and sensing of MPP sensors. **a** Schematic diagram of the preparation process of MXene/PPy in the MPP sponge sensor. **b** Schematic diagram of the piezoresistive sensing mechanism of the MPP sponge. SEM images of the MPP sensor at pressures of 0 kPa (**c**, **f**), 20 kPa (**d**, **g**), and 100 kPa (**e**, **h**)

The problem of improving the pressure-sensitive performance has become the focus issue in next-generation flexible pressure sensors. Our MPP sponge is prepared by dipping-coating the MXene/PPy composite solution and low-temperature drying, and the current collectors are encapsulated on the top and bottom sides of the sponge to complete the device preparation (Fig. 3a). Figs. 3b–e shows the internal skeleton movement and sensitive material contact of the sponge under pressure, where Figs. 3c–h shows SEM images of the MPP sponge at pressures of 0, 20, and 100 kPa. The composite structure of 0D and 2D materials can increase the conductive contact sites and further increase the conductive path inside the sensor. When no pressure is applied, the conductive pathways only exist through the mutual contact of sensitive materials located on the same sponge skeleton. When a pressure is applied, the different sponge skeletons begin to contact each other. The pores inside the sponge gradually shrink. When the pressure is further increased, most of the air inside the sponge is squeezed out by the contact and mutual stacking of the skeletons.

**Fig. 4:**
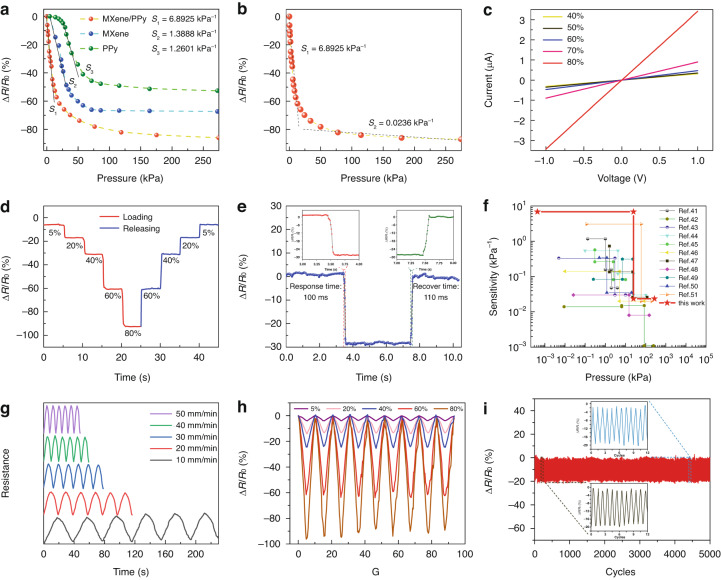
Electrical performance testing of MPP sensors. **a** Comparison graphs of stress sensitivity curves of the MPP sponge, MP sponge, and PP sponge. **b** Calculated pressure sensitivity of MPP sensors. **c** I-V curve with an MPP sponge at 40–80% strain. **d** Resistance response of the MPP sponge from 0–80% step strain. **e** Response/recovery time of MPP sensors at 40% rapid strain. **f** The sensitivity and pressure sensing range of the MPP sensors are compared with those of pressure sensors in the literature. **g** MPP sponge resistance signal at a compression rate of 10–50 mm/min. **h** Cyclic response test graph of the MPP sponge at different strains from 5–80%. **i** Long-term stability of the MPP sponge at 20% strain

A simple circuit is composed by connecting the MPP sensor to a light-emitting diode (LED), as shown in Fig. S6. The LED bulb shines brighter as a finger squeezes the MPP sensor, which occurs due to the decrease in resistance caused by the increase in the internal conductive path during compression in the sensor. The light and dark behavior of the LED bulb prove that the sensor has a negative piezoresistive characteristic. The electrical performance of the MPP sensors was further quantified by first testing the sensitivity performance of five MPP sensors (MPP-1–MPP-5) at different PPy concentrations, and MPP-4 had the best sensing performance (Fig. S7), with subsequent MPP sensors referring specifically to MPP-4. The electrical properties of MPP sponges were examined in more detail, as shown in Fig. 4. The most sensitive pressure regions of the MPP, MP and PP sponges are 0–25, 0–25, and 25–50 kPa, respectively (Fig. 4a). The stress response of the MPP sponge and MP sponge rapidly reaches the maximum sensitivity in the initial stage and then linearly increases until the turnaround point, while the PP sponge presents a resistance hysteresis space. This difference is attributed to the following reasons. Under pressure, the MXene nanosheets that are stacked with each other contact and separate. The contact separation and tunneling effect between the nanoscopic distances create local conductive pathways, which makes the electrical properties of the MPP sponge and MP sponge significantly different from those of the PP sponge. Sensitivity is defined as the variation in relative resistance per unit variation in pressure, which can be written as *S* = Δ*R*/(*R*_0_ × *P*), where Δ*R* denotes the difference between the current resistance and the initial resistance, *R*_0_ denotes the initial resistance, and *P* denotes the current pressure value. *S* determines the sensitivity performance of the pressure sensor; the larger *S* is, the better the sensing performance of the sensor. The maximum sensitivities of the MPP, MP and PP sponges are 6.8925, 1.3888, and 1.2601 kPa^−1^, respectively. This occurs due to the tendency of MXene to overaccumulate and shed, as well as the tendency of its sheet-like structure to crack under small radius bending, hindering electron transfer between MXene nanosheets. Similar to MXene, PPy tends to form random aggregates, leading to its shedding. The combination of MXene and PPy facilitates not only the transfer of electrons but also their good adhesion to the PDMS sponge, improving the properties of the composite through a synergistic effect.

The curve in Fig. 4b shows the variation in the relative resistance of the MPP sponge with pressure. The curve is generally divided into two main parts. The first part is the pressure range of 0–25 kPa, where the sensor sensitivity is 6.8925 kPa^−1^. There are many pores inside the sponge space, and the sponge skeleton collapses rapidly with increasing pressure. The folded MXene nanosheets bond to each other during the collapse process, and the PPy nanoparticles come into contact with each other, resulting in a rapid increase in the conductive pathway inside the sensor, and the sensitivity is significantly higher than that in other stages. The second part is the pressure range of the 25–275 kPa interval with a sensor sensitivity of 0.0236 kPa^−1^. At this stage, most of the air inside the sponge is squeezed out, and the sponge skeletons are tightly bound to each other. Only some of the sensing materials have not yet contacted each other, resulting in a very slow drop in the relative resistance of the sensor. Figure S8 reflects the relationship between the change in relative resistance and pressure for MPP sponges with different porosities, where excessive porosity results in a long contact travel of the conductive material, while a small porosity MPP sponge results in low sensitivity due to the low amount of sensitive material attached to the sponge and severe agglomeration build-up.

Figures S9 and 4c show the variation in the current from −1 V to 1 V for the MPP sponge in the strain states of 0, 5, 10, 20, 30, 40, 50, 60, 70 and 80%. The response is a straight line regardless of the strain state, indicating that the resistance of the sensor remains relatively stable under the applied pressures. Moreover, the slope of the I-V curve increases with increasing strain, which further verifies the sensing mechanism in which the resistance of the MPP sensor decreases with increasing pressure and reflects good linearity. Step strains of 5, 20, 40, 60, and 80% are applied to the MPP sponge (Fig. 4d). The MPP sponge shows excellent discrimination for different strain states. In addition, the resistance response under the released pressure state is basically the same as that under the applied pressure state, which broadens the application scenario of the sensor. Figure 4e shows the instantaneous 30% strain applied to the MPP sensor, where the response time and recovery time are 100 and 110 ms, respectively. Figure 4f compares the sensitivity and pressure sensing range of the MPP sponge sensor with those of pressure sensors in the literature^41–51^. Prior literature data include nine sponge-type^41–45,47–50^ and two nonsponge-type sensors^46,51^. Our MPP sensors demonstrate some competitive strengths in terms of sensitivity and sensing range, particularly in low pressure detection performance. The cyclic response behavior of the MPP sponge at different frequencies is investigated. As shown in Figs. 4g and S10, the compression speed of the tensile machine at 40% fixed strain is set to 10, 20, 30, 40, 50, 100, 150, 200 and 300 mm/min to adjust the frequency. At the same strain and different compression frequencies, the resistance response remains stable, and the curve shapes all remain consistent, which indicates the favorable frequency stability of the MPP sponge. The MPP sponge is subjected to pressure cycling tests at different strains of 5, 20, 40, 60, and 80% (Fig. 4h). The signal shape and peak value of the device remain constant under different strains, and the variation in resistance has good repeatability. In addition, the MPP sensor was tested for 5000 continuous compression-release cycles (Fig. 4i). The sensor still has an extremely stable signal output, which effectively proves the excellent long-term stability of the MPP sponge and provides stability assurance for its application in human motion. To explore the minimum detectable pressure of the MPP sponge, a polyurethane sponge (0.01 g) is placed on the MPP sensor to assess the change in resistance of the device, as shown in Fig. S11. The sensor produces a significant response when the polyurethane sponge is placed and removed at 2 and 8 s, indicating that the minimum detection limit of the MPP sensor is lower than 0.43 Pa.

### MPP sensor for human motion detection

**Fig. 5:**
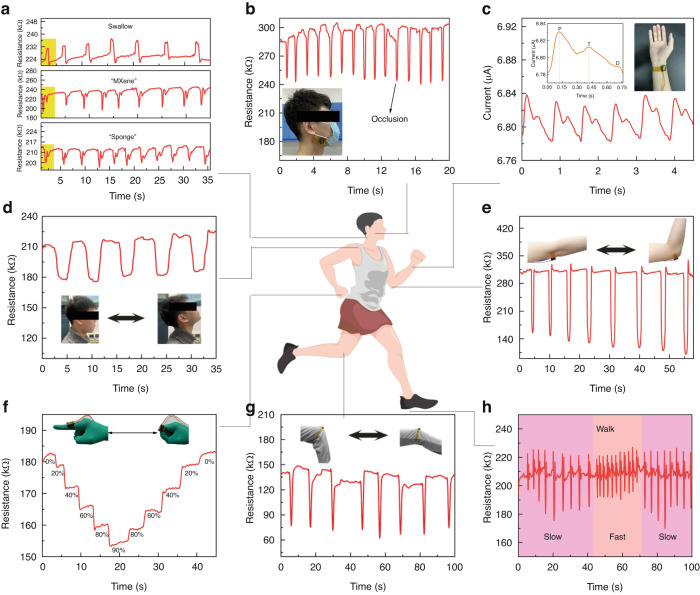
Application of MPP sensors in human movement. **a** MPP sensor resistance signal during pharyngeal movement (swallowing action, “MXene” and “sponge” pronunciation). **b** MPP sensor resistance signal at the occlusal muscle during occlusal action. **c** Pulse signal from the MPP sensor measuring the human radial artery. **d** MPP sensor resistance signal at the back of the neck when tilting the head. **e** MPP sensor resistance at the elbow position during elbow flexion. **f** MPP sensor resistance signal at the knuckle area when bending fingers at different angles. **g** MPP sensor resistance signal at the knee area during leg flexion. **h** MPP sensor resistance signal at the heel area during walking and running

The MPP sponge sensor can detect human motion based on its high sensitivity and stability. The sensors are attached to different parts of the body to detect their activity during human movement. The resistance changes of the pressure sensor with different movements are shown in Fig. 5. The MPP sensor can detect not only small human movements, such as index finger activity during mouse clicks and double clicks (Fig. S12); throat vibrations when swallowing and uttering the words “MXene” and “sponge” (Fig. 5a); and the force of occlusal muscles (Fig. 5b). It can even detect the tiny pulse signal of the human radial artery (Fig. 5c) and a larger range of human articular movements, such as head tilt (Fig. 5d), elbow bend (Fig. 5e), finger bend at different angles (0, 20°, 40°, 60°, 80°, 90°) (Fig. 5f), knee bending (Fig. 5g), and walking state (Fig. 5h).

In the pharyngeal activity test, the MPP sensor shows a single characteristic peak when swallowing and two peaks with differences when spelling “MXene” and “sponge”, indicating that the MPP sensor can distinguish different pharyngeal activities. The device resistance response is nearly consistent with good repeatability when swallowing and spelling words repeatedly. In the mouse single- and double-click tests, single peaks (tester clicks the mouse) and double peaks (tester double-clicks the mouse) can be clearly determined. Therefore, the MPP sponge sensor can be employed to detect the single or double mouse clicks of the tester. In the radial artery pulse test, the MPP sensor detects the P, T and D peaks in the pulse signal. The ratio of peak width to peak height between the PT and TD is analyzed to provide a preliminary assessment of the cardiovascular status. In the human walking condition test, the frequency of the signal is used to identify the movement of the tester, with the signal frequency being lower when walking and higher when running. In summary, MPP sponge pressure sensors have been proven to have promising applications in the field of body detection.

Wearable sensors need to exhibit strong environmental adaptability in practical applications. Figure S13a, b evaluates the pressure and response of the MPP sponge sensor at 50% strain after washing in deionized water for 1 h compared with before washing. The pressure, resistance waveform and peak value did not change significantly. Figure S13c, d shows the changes in the resistance of knee bending and finger bending before and after washing the MPP sensor with deionized water. The signal response of the sensor after washing is essentially the same as that before washing, except that the deionized water increases the resistance of the device. This excellent environmental adaptability is attributed to the stable combination of MXene and PPy.

### Human fitness movement posture recognition

**Fig. 6:**
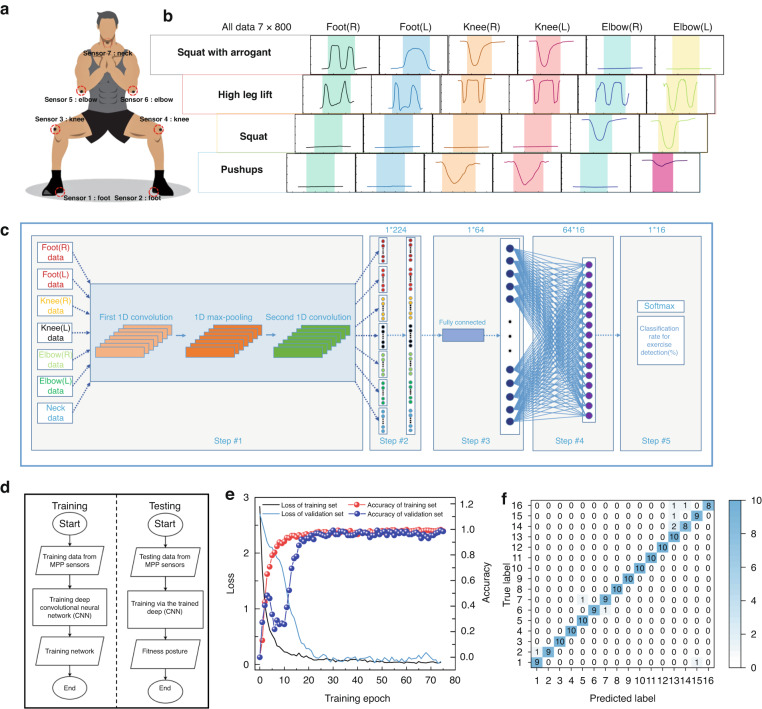
The MPP sensor-based human fitness posture recognition system assisted by the CNN deep learning algorithm. **a** Diagram of seven MPP sensors placed on human joints. **b** Graphs of MPP sensor resistance signals at different parts of arrow squat, high lift, deep squat and push-up movements. **c** Schematic diagram of data training and recognition based on a multi-input CNN neural network. **d** Flow chart of the pose recognition system based on the MPP sensor. **e** Confusion matrix of the multi-input CNN neural network model. **f** Accuracy and loss curves of the training set and test set under different training epochs

Recently, deep learning has been increasingly researched in combination with other fields because of its advantages of high-level learning ability, wide coverage, adaptability, and resistance to interference^52–54^. Seven identical MPP sponge pressure sensors were prepared and fixed to the heels, knees, elbows, and skin on the back of the neck (Fig. 6a) of the tester (a healthy adult male) to detect joint movement or contact with the ground. The MPP sensors can detect specific signals from different areas of activity when the tester performs four fitness movements: deep squat, push-up, high leg lift, and arrow squat (Fig. 6b). When performing deep squatting, for example, the part of the sensor excluding that touching the knee joint does not significantly move, so only two MPP sensors in the knee area produce clear signals. The above results demonstrate that the MPP sensor can distinguish the activity of different parts. To further validate the potential applications of MPP sensors in fitness posture recognition, a multi-input CNN deep learning algorithm was constructed to train and analyze the data generated by MPP sensors. To ensure that the deep learning algorithm can fully learn the characteristics of different fitness postures and reduce the influence of human factors and systematic noise of the sampling equipment, 800 repetitions of sampling were performed for the seven corresponding sensor response changes to four movements: deep squats, push-ups, high leg raises, and arrow squats. Each of the actions is configured with one correct action and three incorrect actions, totaling 16 actions (Figs. S14–S17). The waveforms of the same action show similar characteristic peaks, indicating that the sensor has excellent reliability.

A multi-input deep convolutional neural network (CNN) deep learning algorithm was applied to model the sampled data after feature normalization. The basic structure of the multi-input deep CNN in this work is shown in Fig. 6c. In a ratio of 4:1 training set to test set, 640 randomly selected resistance response data from the fitness posture database were used as the training set to train the CNN model. The remaining 160 data points were used as the test set to verify the final recognition ability of the model. The data are normalized before being imported into the input layer. The addition of a convolutional layer to increase the depth of the neural network improves the network performance. Table 1 summarizes the hyperparameters. Each convolution is followed by a ReLu activation function to prevent the gradient from exploding and disappearing. The first convolution contains 8 convolutional kernels with a size of 8 × 1 and a stride of 1. The number of convolutional kernels in the second convolution is 16, and the other parameters remain unchanged. In addition, the size of the maximum pooling windows is 2 × 2 with a stride of 2. The optimizer of the CNN model is Adam. The combination of values for the hyperparameters is determined by using a grid search.

**Table 1:** Hyperparameters of the multi-CNN model

Hyperparameters	Values	Hyperparameters	Values
The number of neurons in first convolution	32	MaxEpochs	75
The number of neurons in second convolution	16	Initial learn rate	0.2
The number of neurons in Dense1	32	ReLu	0.2
The number of neurons in Dense2	64	MaxPooling	2

Deep learning models require continuous learning and tuning to obtain optimal performance^54,55^. First, the multi-input CNN model is trained through a large amount of measurement data from different fitness movements. The models are then trained iteratively using the errors on the dataset to obtain an appropriate model that fits the dataset. Figure 6d depicts the basic architecture of the training and testing system, including data acquisition, feature extraction, and target identification classification. In the training phase, the training data obtained from MPP sensors are normalized and input into the CNN deep learning algorithm for fitness posture training. Finally, the recognition accuracy of the test set reaches 95% (Fig. 6f). This demonstrates the feasibility of CNN deep learning algorithms to assist MPP pressure sensors in human motion posture recognition and the enormous potential of MPP sensors for human-computer interaction and intelligent sensing applications.

## Conclusion

In summary, MPP sensors were constructed through the preparation of PDMS sponges by the sugar sacrificial template method and an MXene/PPy composite solution by in situ polymerization. The PDMS sponge enhances the flexibility and stability of the sensor. The addition of PPy and MXene provides the sensor with excellent electrical conductivity, mechanical properties, repeatability and sensitivity (6.8925 kPa^−1^) over the 0–15 kPa excitation range. The MPP sensor achieves maximum sensitivity at the initial pressure stage, which gives the sensor excellent micropressure monitoring performance. The MXene and PPy adhere more stably to the surface of the sponge skeleton through strong intermolecular forces, ensuring that the conductive sponge is wash-resistant and maintains excellent reproducibility under different degrees of repeated testing. With its excellent mechanical and conductive properties, the MPP sponge can detect human movement in a variety of situations, including head lifting, swallowing, finger bending, elbow bending and knee bending. In addition, the MPP sensor combined with deep learning methods achieves a 95% correct recognition rate for different human movement postures, which proves the potential value of the sensor in practical applications. In conclusion, MPP sponge sensors offer great potential for applications in smart wearable devices and human movement detection.

### Supplementary information

Supplementary Information
