# Supplementary material for: MXene/PPy@PDMS sponge-based flexible pressure sensor for human posture recognition with the assistance of a convolutional neural network in deep learning
Source: Microsyst Nanoeng. 2023 Dec 19;9:155. doi: 10.1038/s41378-023-00605-0 (PMC10728160; doi:10.1038/s41378-023-00605-0)
Supplement: Supplementary file 1 — Supplementary Information [file 41378_2023_605_MOESM1_ESM.docx]

**Supporting Information**

**MXene/PPy@PDMS sponge-based flexible pressure sensor for human posture recognition with the assistance of convolutional neural network in deep-learning**

**Hui Xia ^a, #^, Lin Wang ^b, #^, Hao Zhang ^a^, Zihu Wang ^a^, Liang Zhu ^b^, Haolin Cai ^a^, Yanhua Ma ^a^, Zhe Yang ^b,*^, Dongzhi Zhang ^a,^***

^a^ College of Control Science and Engineering, China University of Petroleum (East China), Qingdao 266580, China

^b^ State Key Laboratory of Chemical Safety, SINOPEC Research Institute of Safety Engineering Co., Ltd, Qingdao 266071, China

# These authors contributed equally to this work.

*Corresponding author: Dongzhi Zhang, Zhe Yang

E-mail address: [dzzhang@upc.edu.cn](mailto:dzzhang@upc.edu.cn), yangzhe.qday@sinopec.com

Tel: +86-532-86982928

Fax: +86-532-86981335


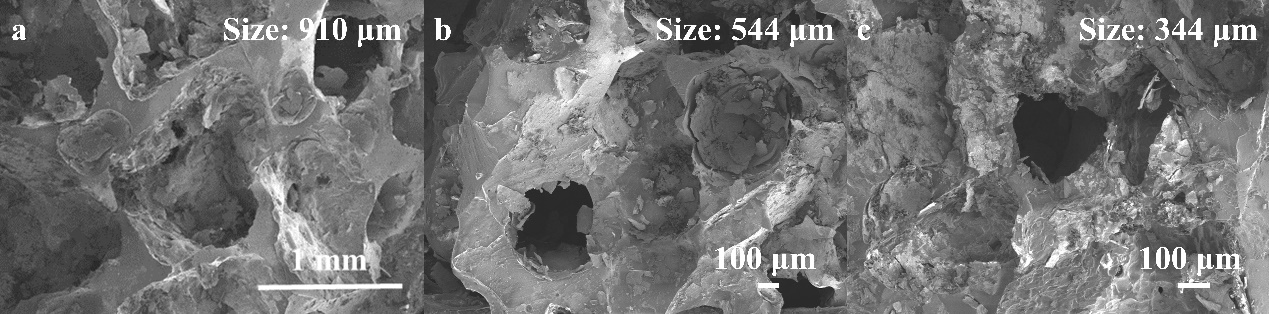


**Figure S1** SEM images of MPP sponges with different porosity.


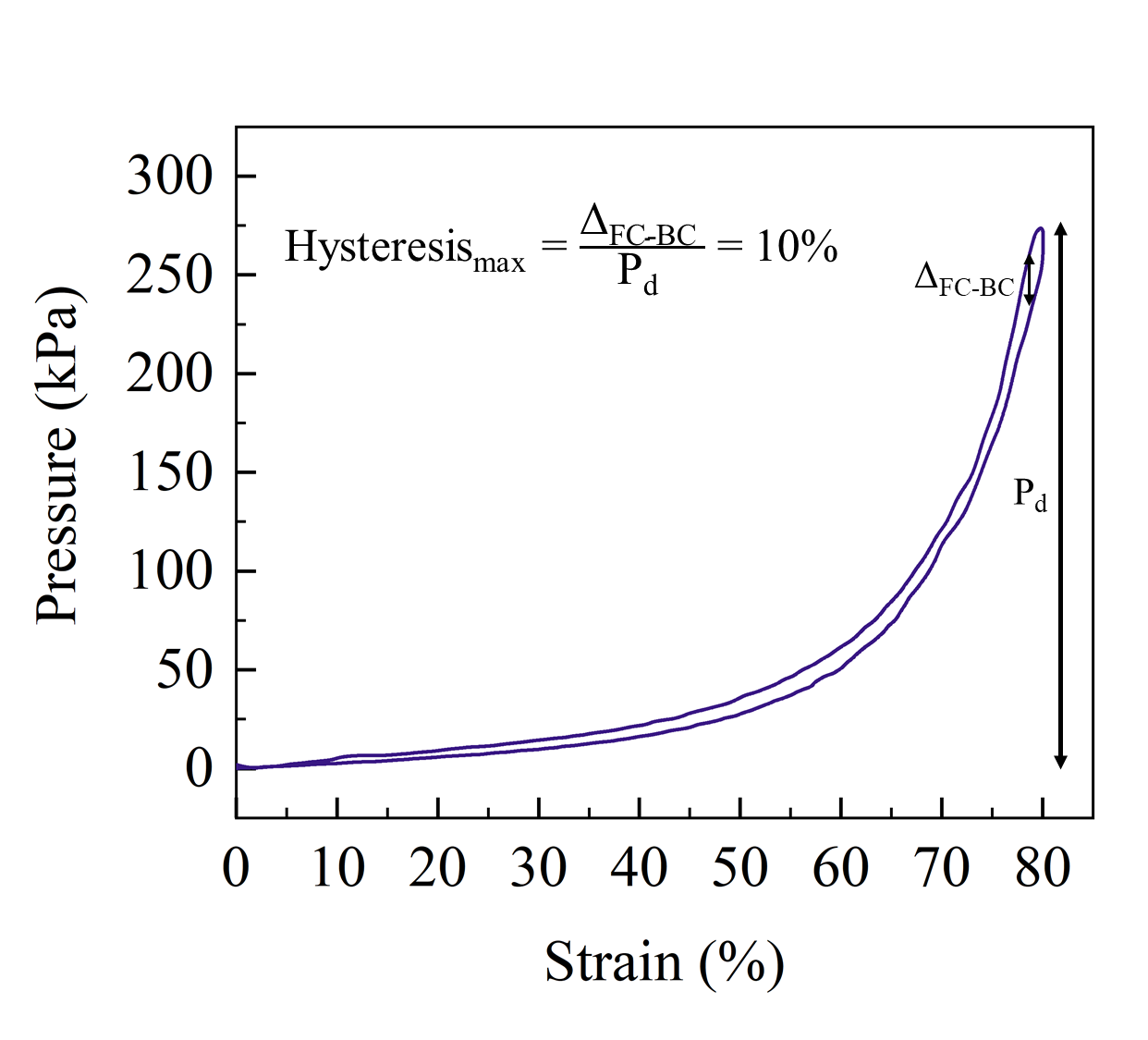


**Figure S2** The MXene/CB@PU sensor current response under continuous identical pressure loading and unloading.


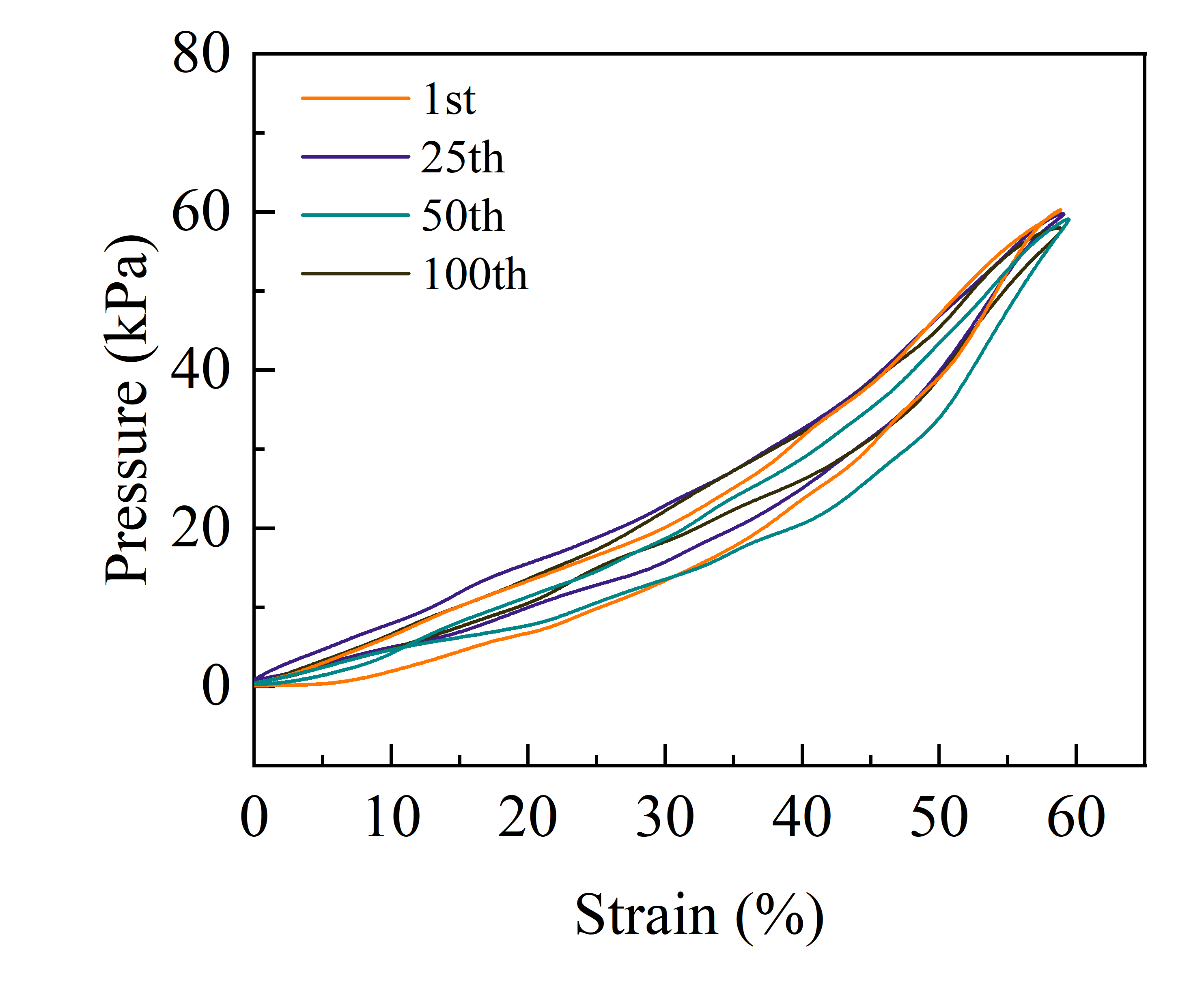


**Figure S3** 1-100 repetitions stress-strain cyclic curves of MPP sponge.


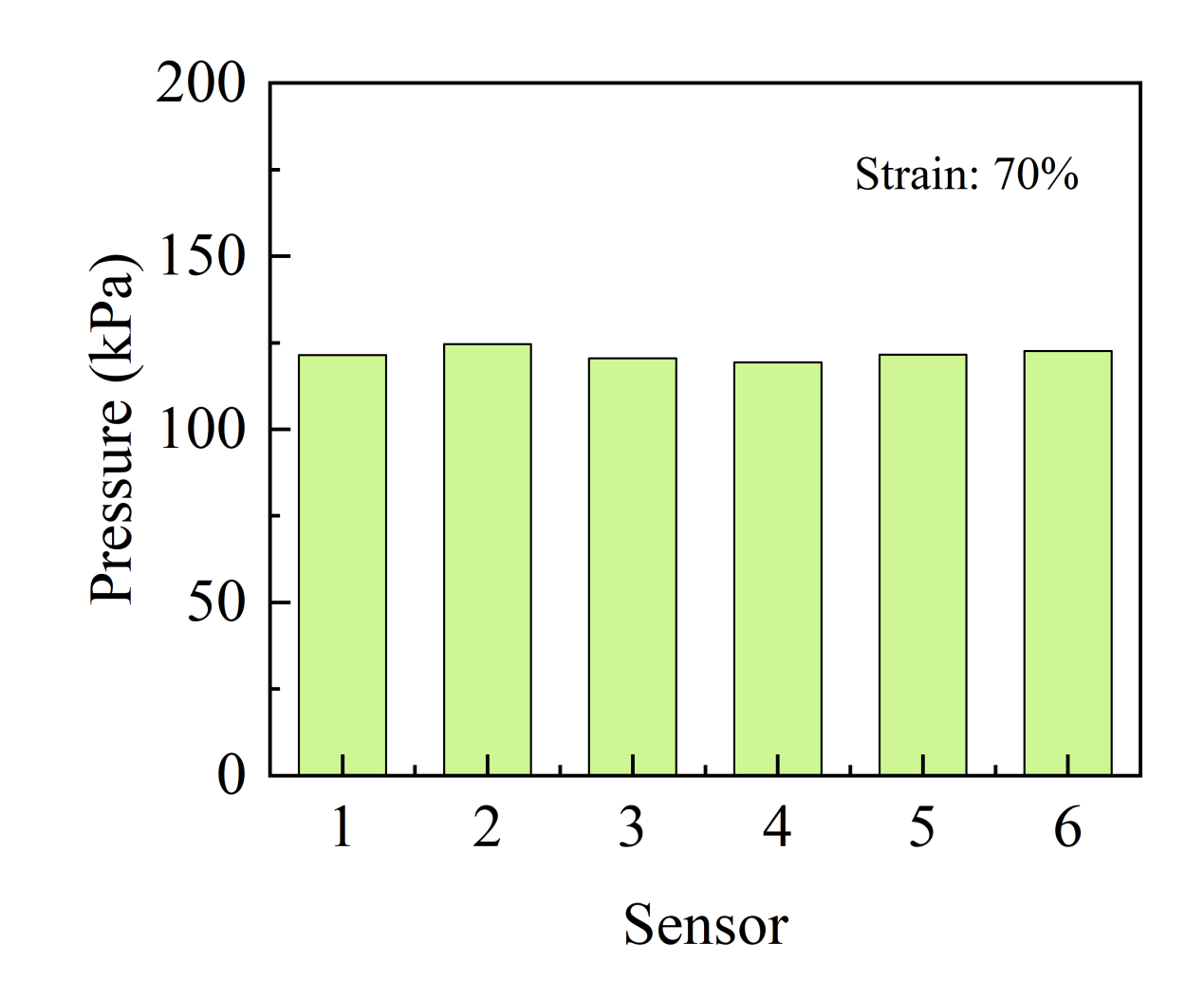


**Figure S4** Stress at 70% strain for six MPP sponges of the same preparation method.


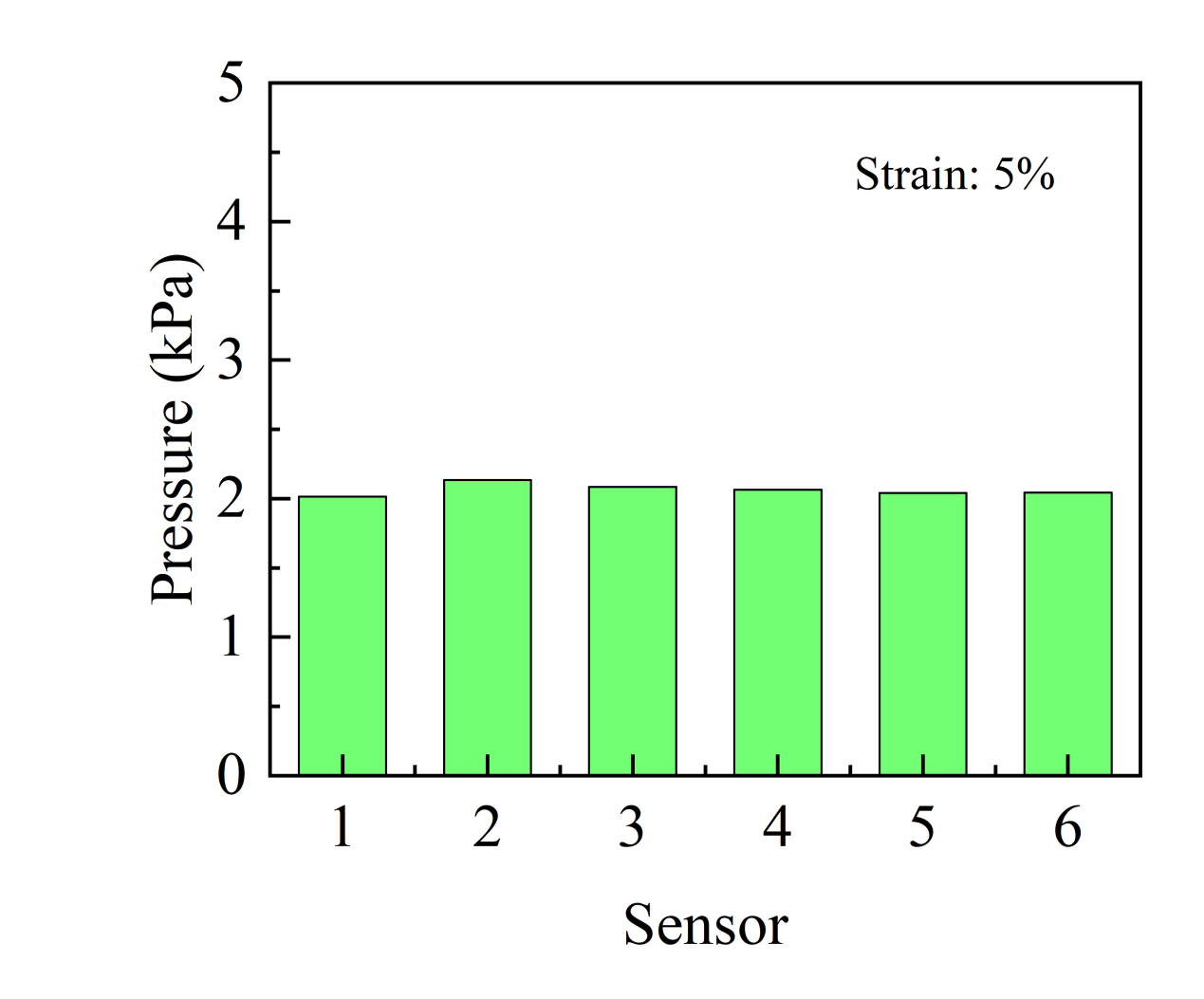


**Figure S5** Stress at 5% strain for six MPP sponges of the same preparation method.


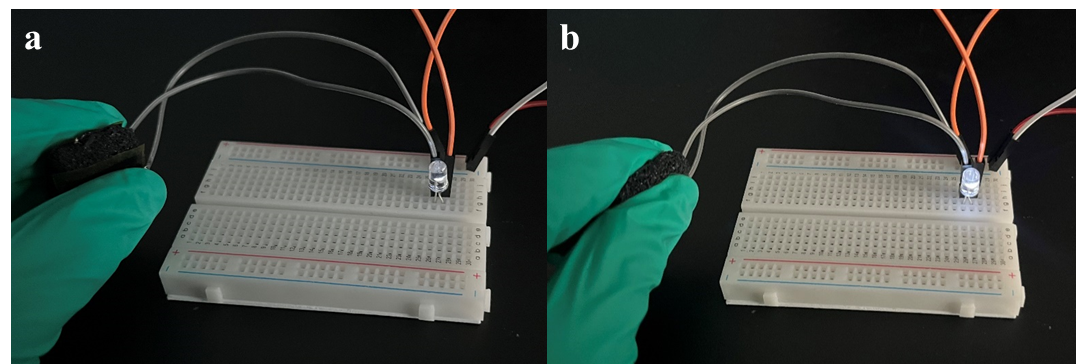


**Figure S6** Photograph of MPP sponge lighting LED bulb in initial and compressed state.


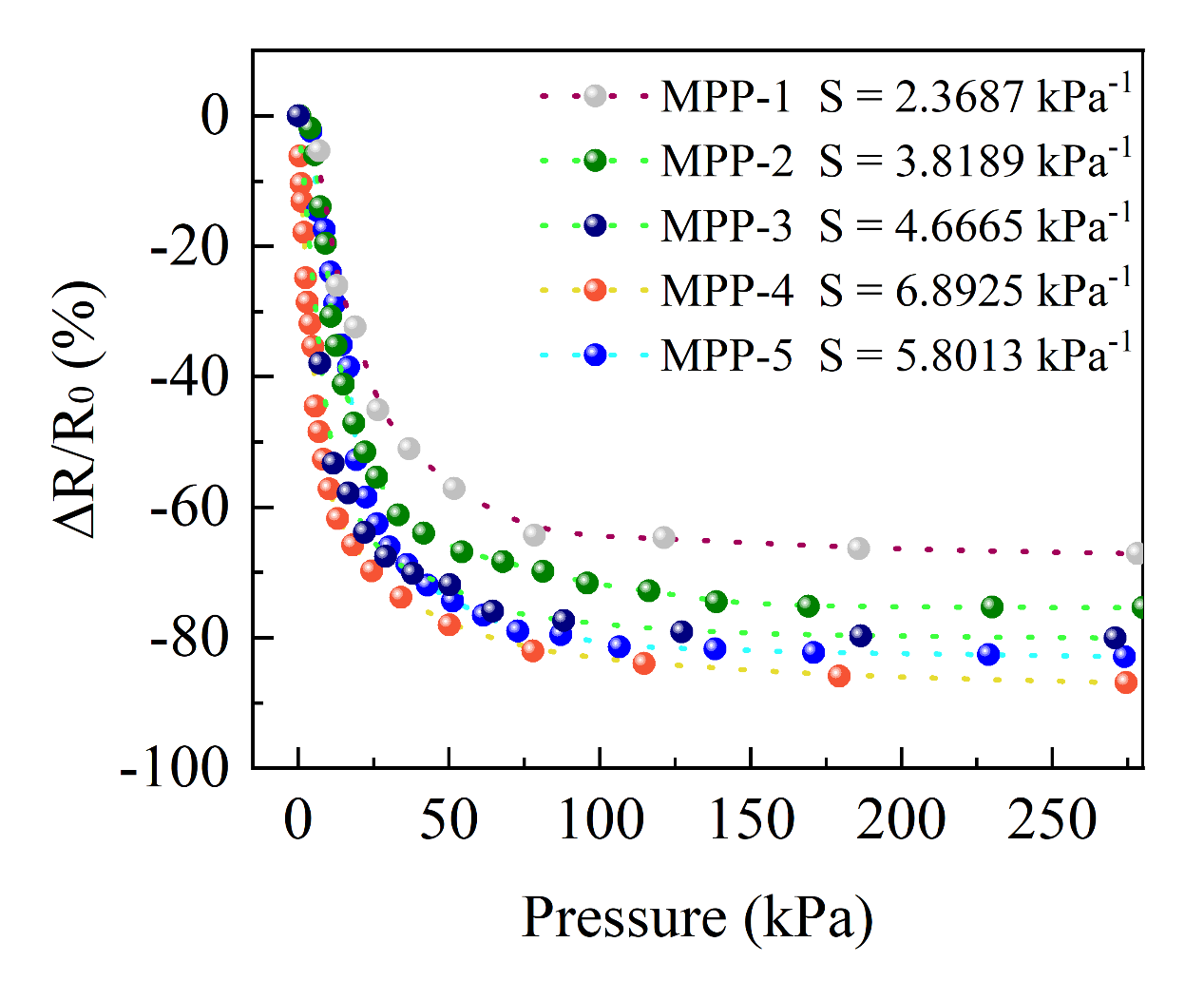


**Figure S7** Sensitivity properties of MPP sponges with different MXene and PPy ratios.


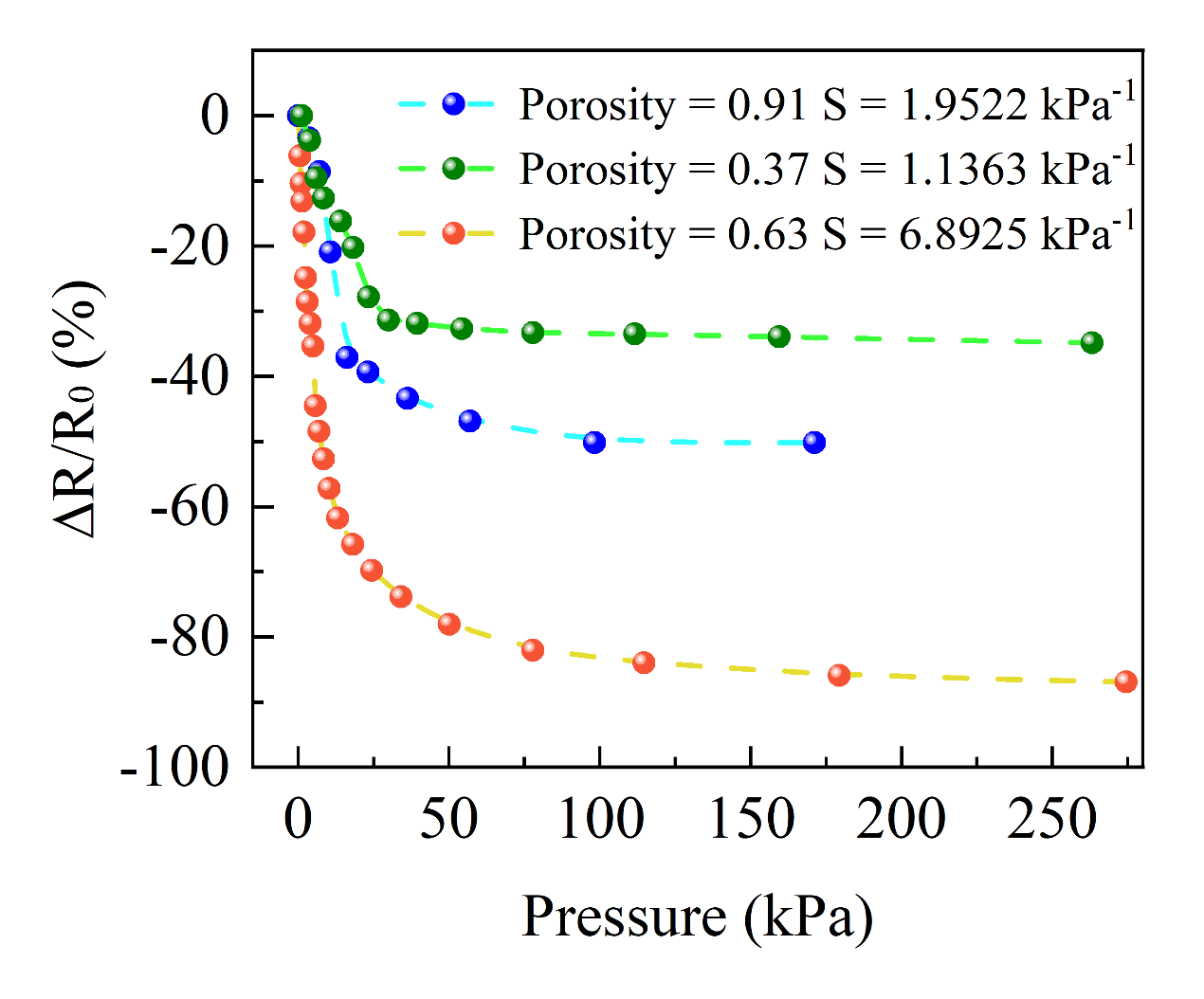


**Figure S8** Sensitivity properties of MPP sponges with different porosities.


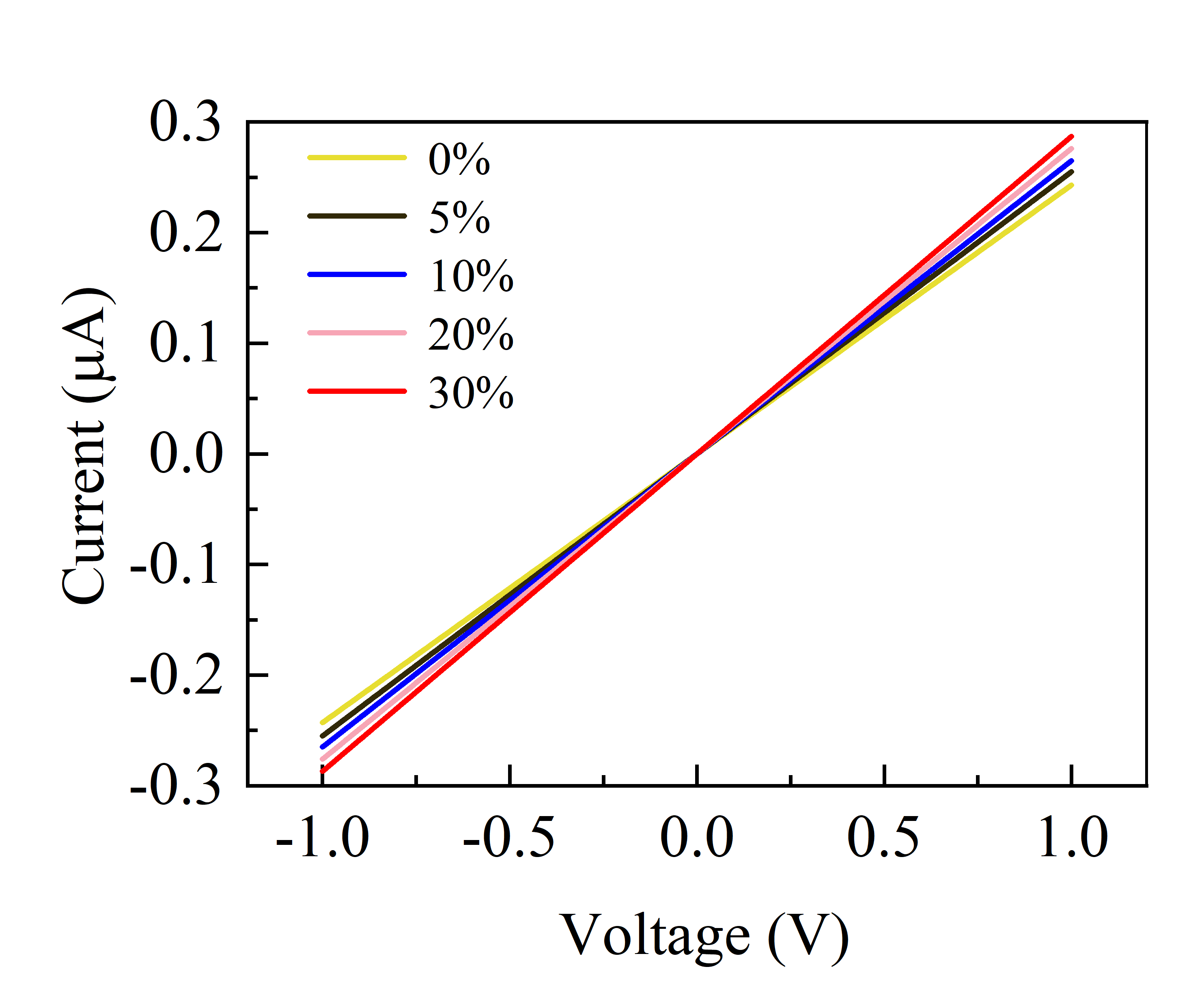


**Figure S9** I-V curves of MPP sponge at the strain of 0, 5%, 10%, 20%, 30%.


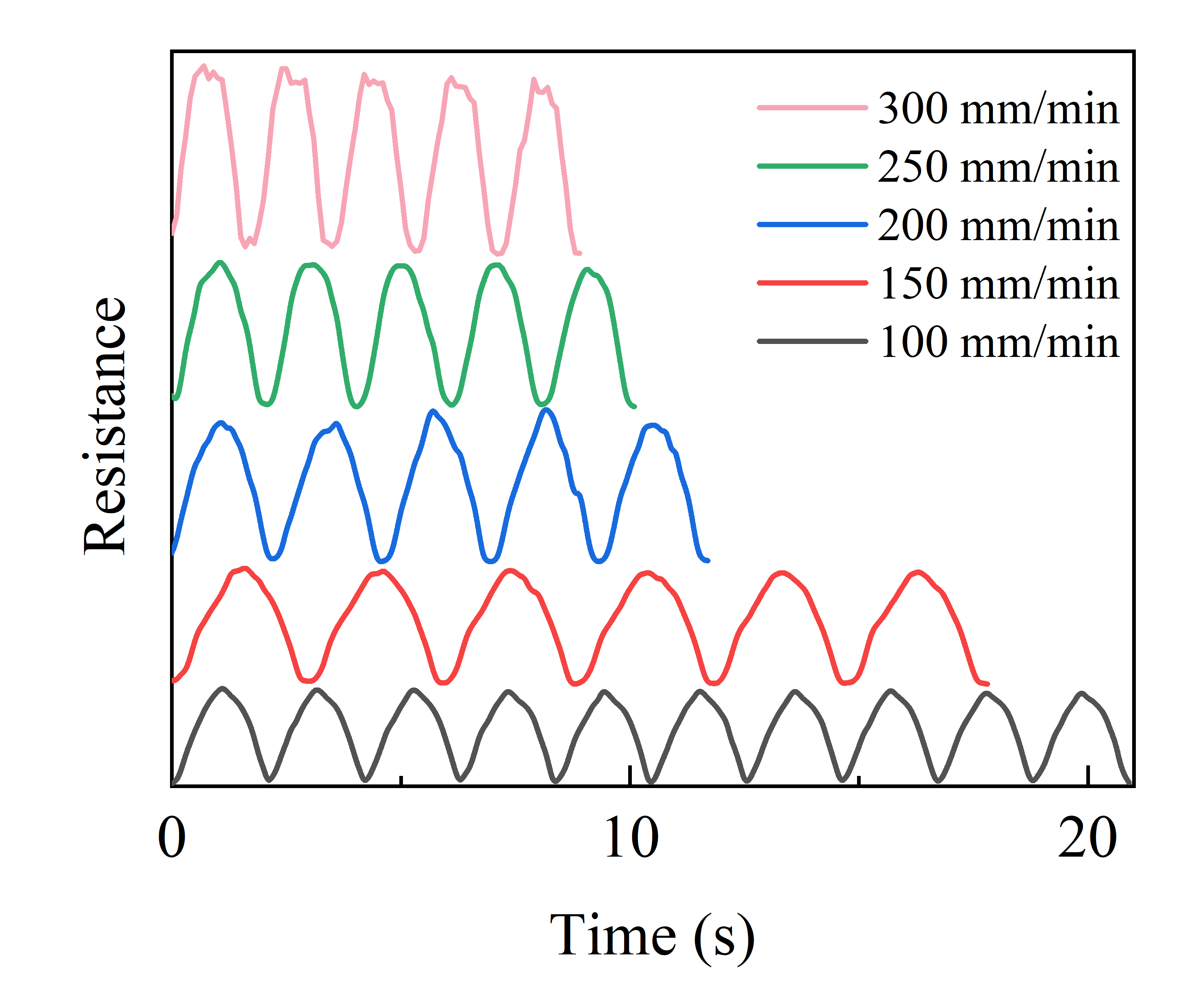


**Figure S10** The variation curve in resistance value of MPP sponge at stress speed of 100 mm/min, 150 mm/min, 200 mm/min, 250 mm/min, 300 mm/min.


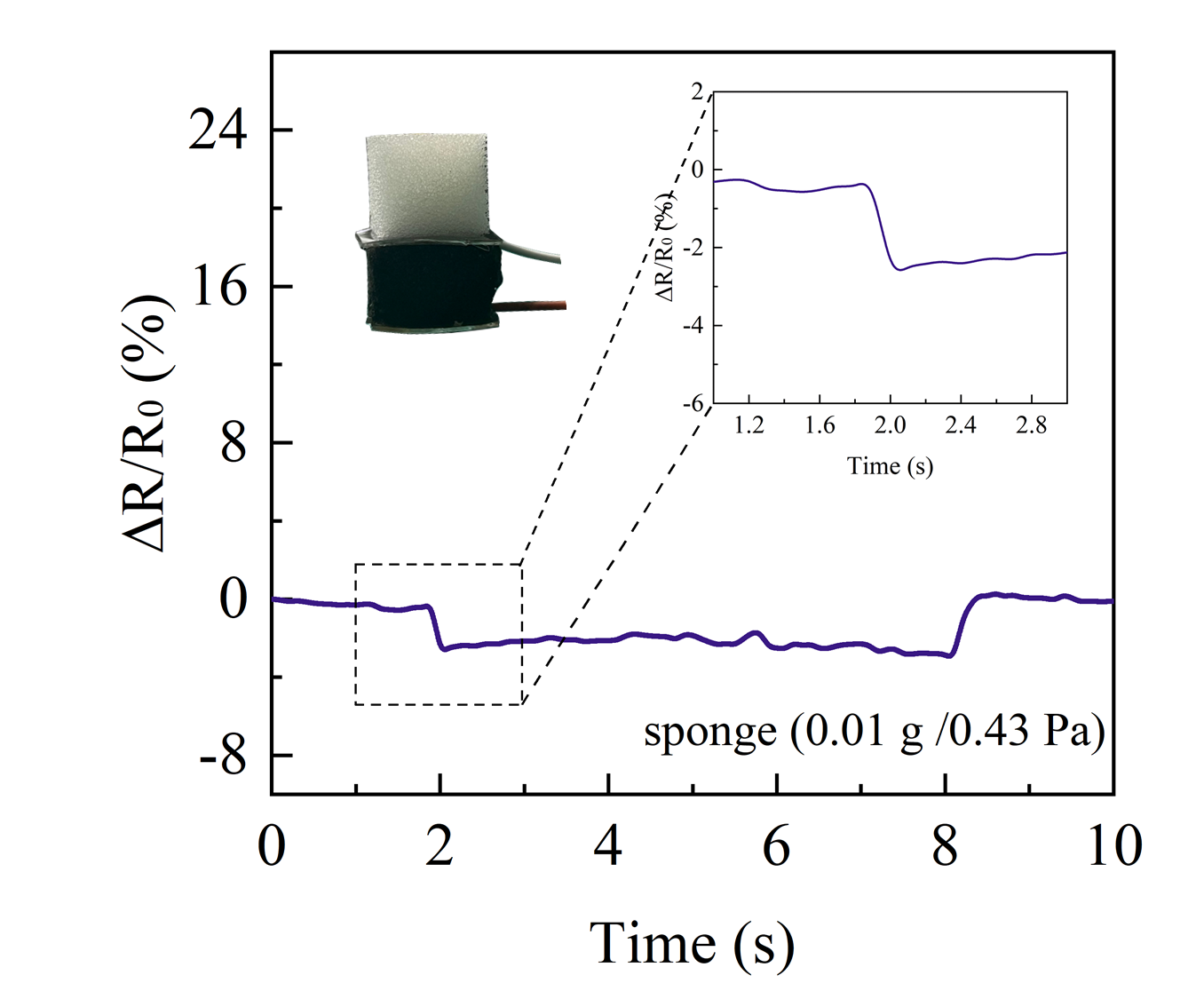


**Figure S11** The resistance response of 0.43 Pa polyurethane sponge placed on MPP sponge.


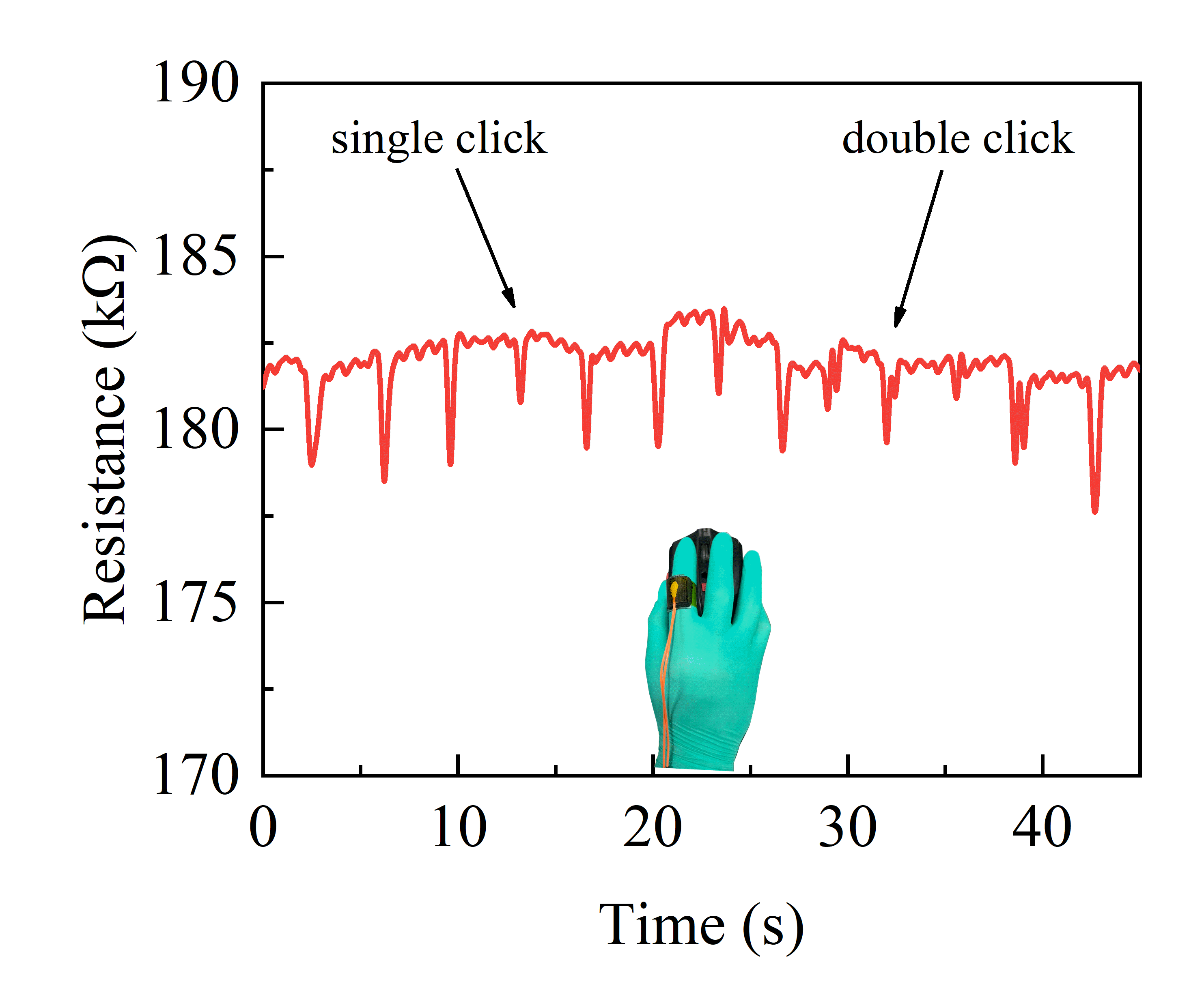


**Figure S12** The variation of the resistance on the MPP sensor fixed to the index finger during single and double clicks of the hand-held mouse.


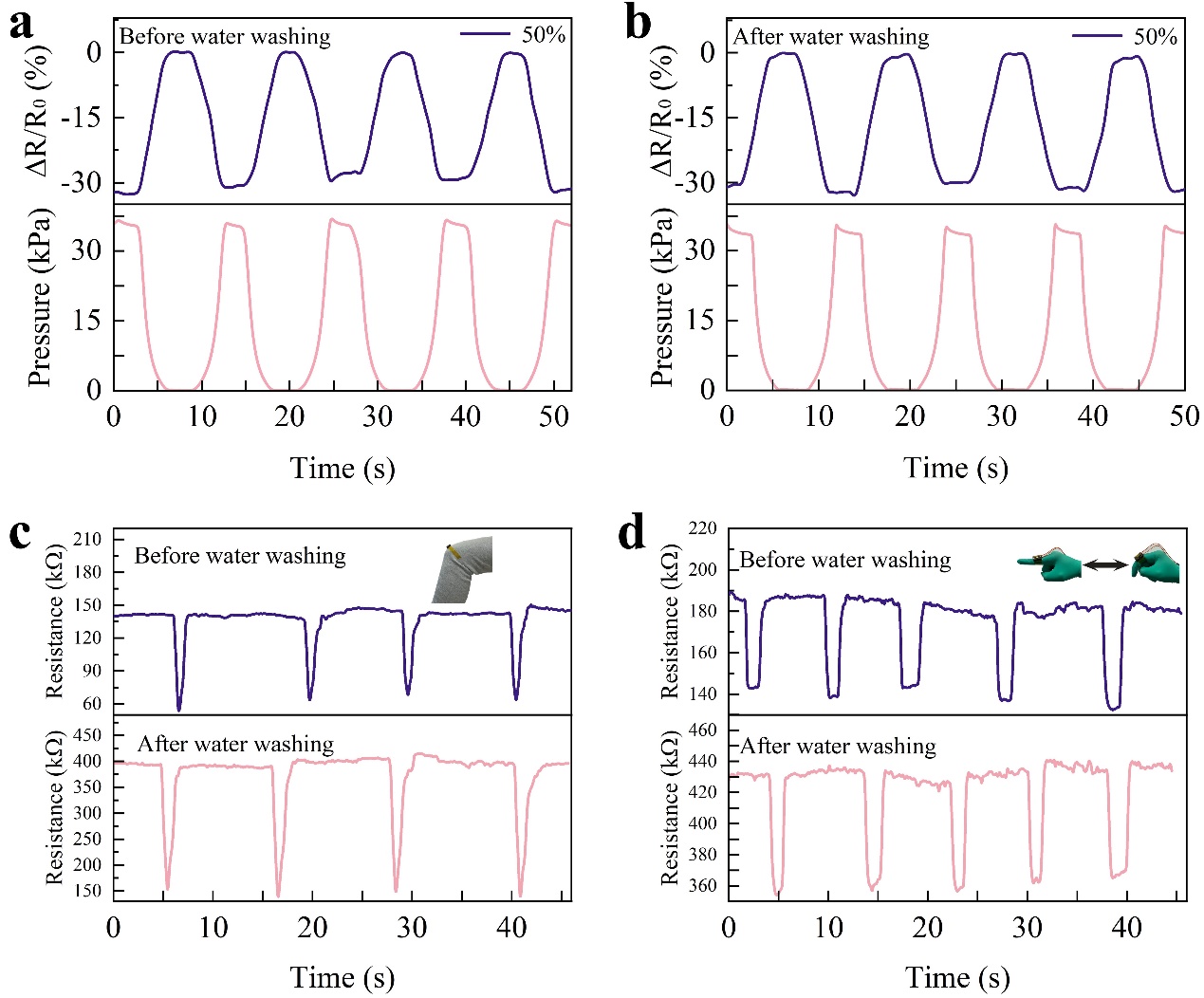


**Figure S13** **Wash-resistance test for MPP sensors** **a** Resistance response and stress of MPP sensor at 50% strain before water washing, **b** Resistance response and stress of MPP sensor at 50% strain after water washing, **c** MPP sensor detecting knee bending before and after water washing, **d** MPP sensor detecting finger bending before and after water washing.


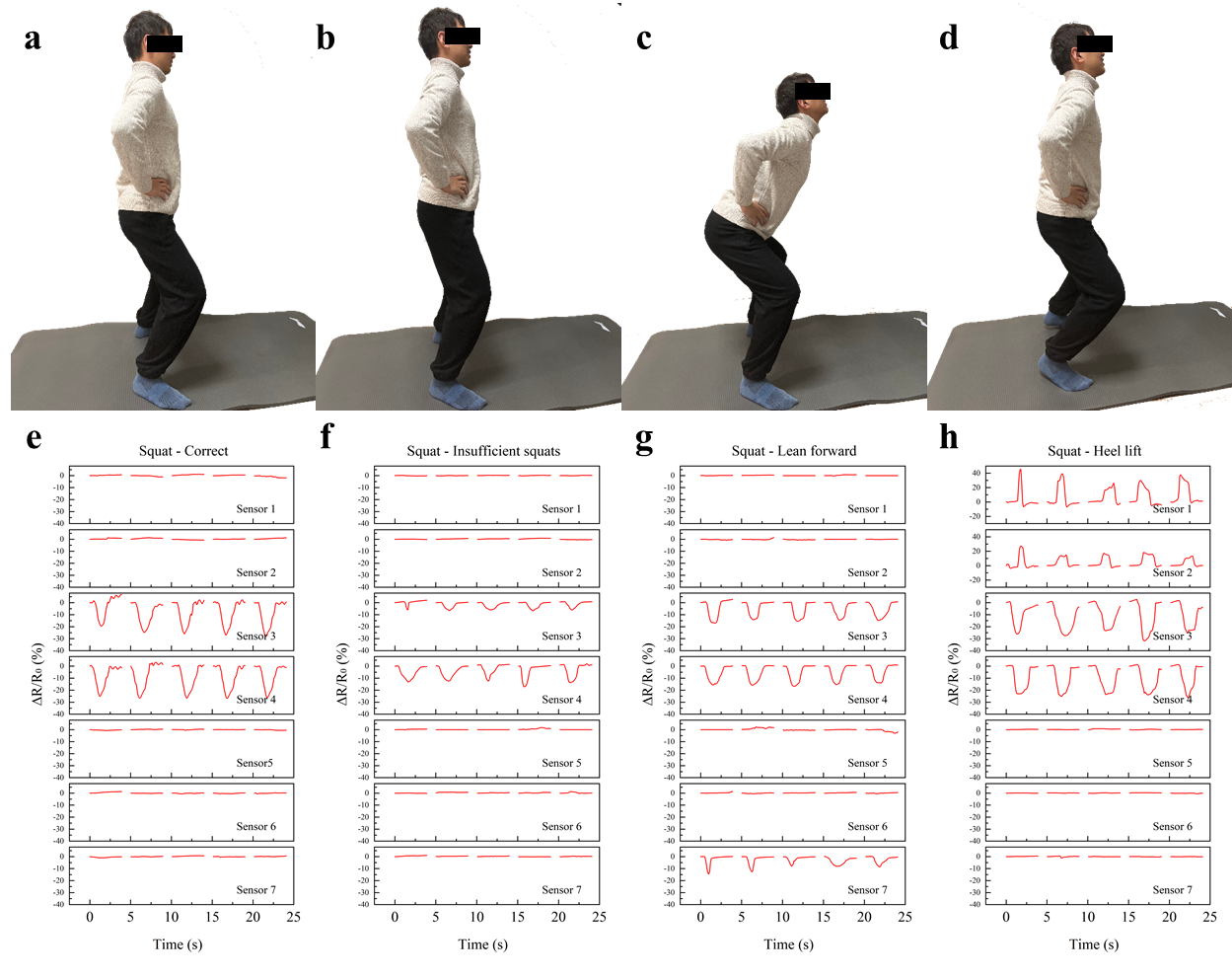


**Figure S14 Fitness posture: squat.** **a-d** Diagram of the correct, insufficient squat, lean forward and heel lift position. **e-h** The signal of MPP sensor during correct, insufficient squat, lean forward and heel lift position.


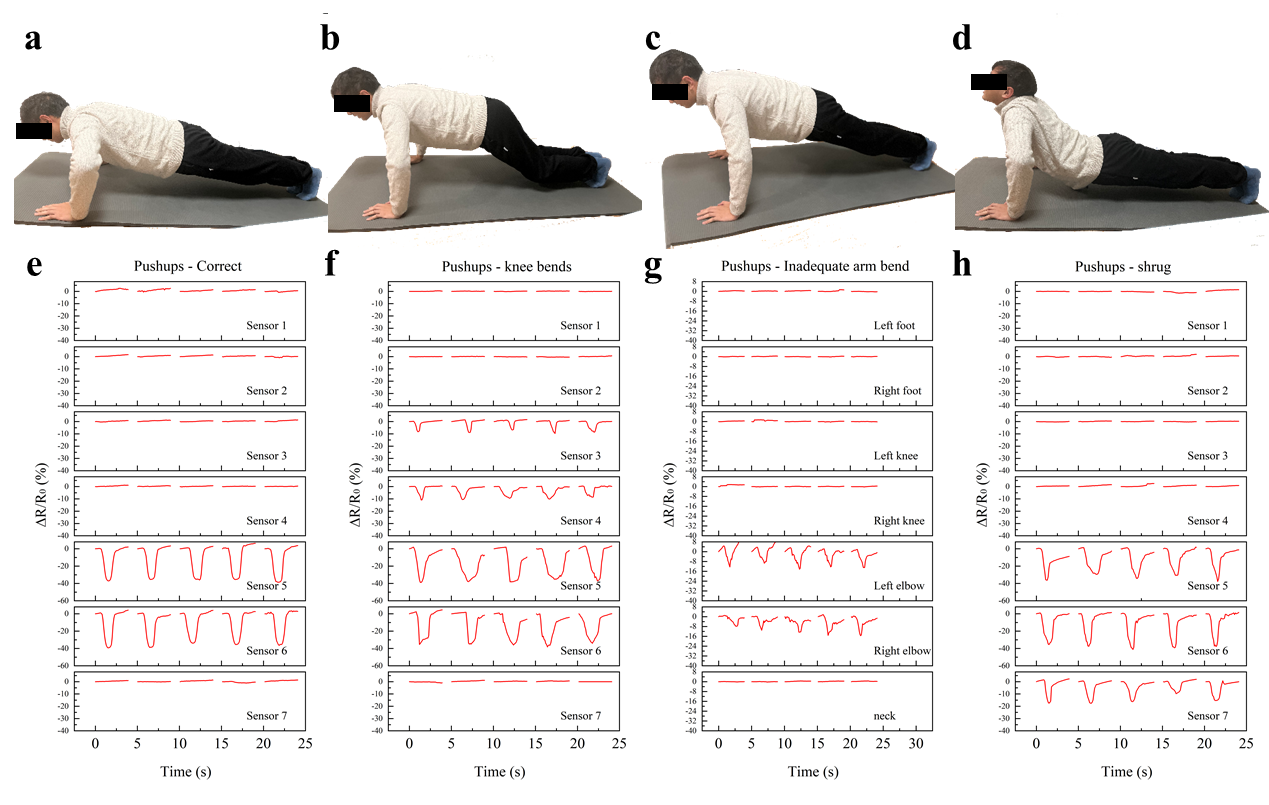


**Figure S15** **Fitness posture: pushups.** **a-d** Diagram of the correct, knee bends, inadequate arm bend, shrug position. **e-h** Graph of MPP sensor signal during correct, knee bends, inadequate arm bend, shrug position.


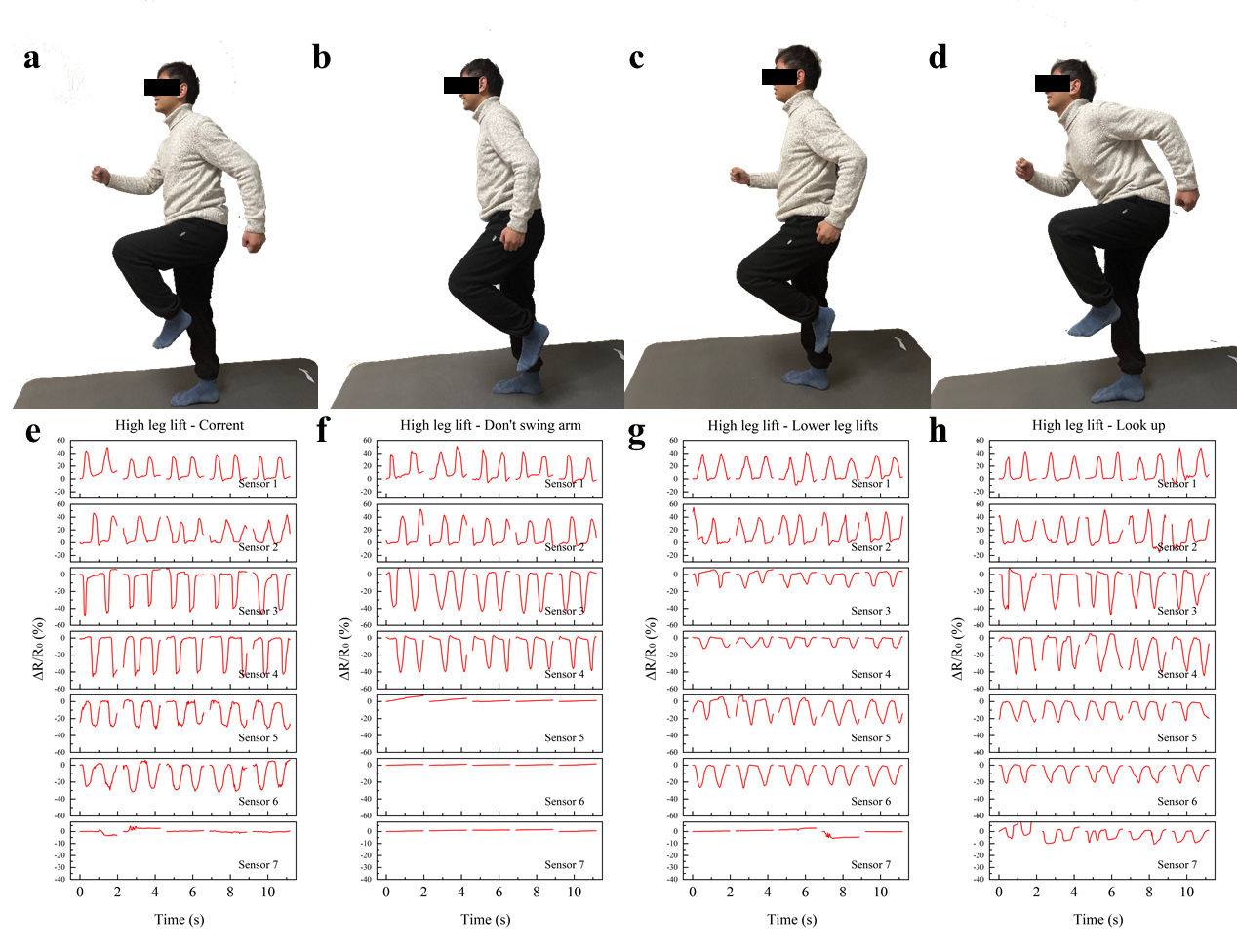


**Figure S16** **Fitness posture: high leg lift.** **a-d** Diagram of the correct, don’t swing arm, lower leg lifts, look up position. **e-h** Graph of MPP sensor signal during correct, don’t swing arm, lower leg lifts, look up position.


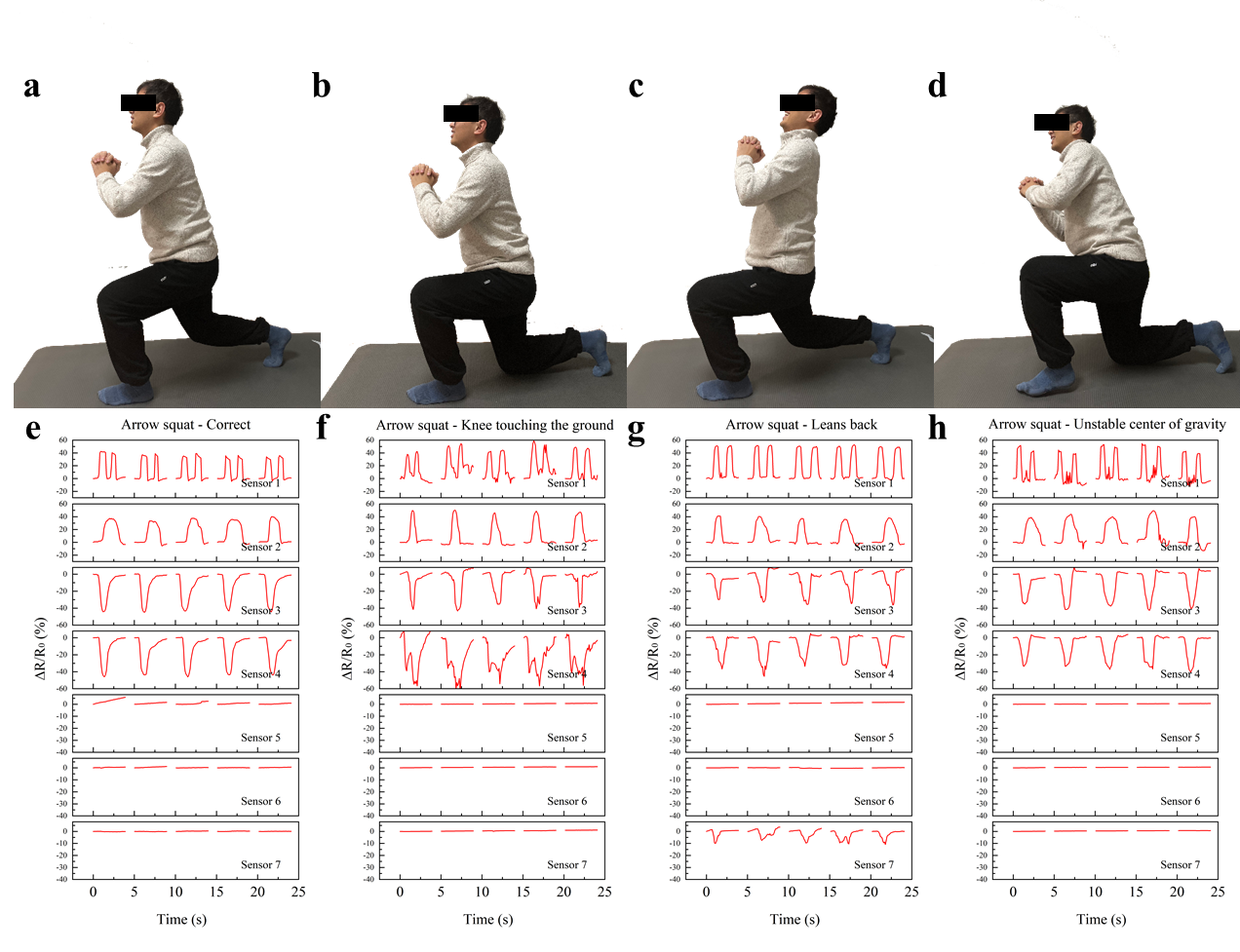


**Figure S17 Fitness posture: arrow squat.** **a-d** Diagram of the correct, knee touching the ground, leans back, unstable center of gravity position. **e-h** Graph of MPP sensor signal during correct, knee touching the ground, leans back, unstable center of gravity position.
